# Ex-situ X-ray computed tomography, tension clamp and in-situ transilluminated white light imaging data of non-crimp fabric based fibre composite under fatigue loading

**DOI:** 10.1016/j.dib.2018.09.109

**Published:** 2018-10-03

**Authors:** Kristine M. Jespersen, Jens A. Glud, Jens Zangenberg, Atsushi Hosoi, Hiroyuki Kawada, Lars P. Mikkelsen

**Affiliations:** a Department of Wind Energy, Section of Composites and Materials Mechanics, Technical University of Denmark, Fredriksborgvej 399, 4000 Roskilde, Denmark; b Department of Mechanical and Manufacturing Engineering, Aalborg University, Fibigerstraede 16, 9220 Aalborg Oest, Denmark; c LM Wind Power Blades, Composite Mechanics, Jupitervej 6, 6000 Kolding, Denmark; d Department of Applied Mechanics and Aerospace Engineering, Waseda University, 3-4-1, Okubo, Shinjuku-ku, Tokyo 169-8555, Japan

## Abstract

The data published with this paper is obtained during fatigue testing of a unidirectional non-crimp fabric based glass fibre composite by means of ex-situ X-ray CT and in-situ transilluminated white light imaging experiments. The data experimentally shows the damage initiation and progression under fatigue loading both in terms of off-axis cracks in the thin supporting backing fibre bundles and fibre fractures in the load carrying fibre bundles. X-ray CT data comparing the loaded and unloaded state of damage regions by means of a tension clamp solution are also published with this paper.

**Specifications table**

**Table:** 

Subject area	*Physics*
More specific subject area	*Fibre composites, Damage mechanics*
Type of data	*Image (X-ray computed tomography data sets and high-resolution photos)*
How data was acquired	*Zeiss Xradia Versa 520 (X-ray CT) and Nikon D7000 camera with a Tokina Macro 100 F2.8D lens*
Data format	*Reconstructed CT data, Raw photo data, videos*
Experimental factors	*Interrupted tension-tension fatigue test (R=0.1)*
Experimental features	*High resolution X-ray CT scans performed in the same region after each interruption point of the fatigue test (ex-situ X-ray CT fatigue test) and X-ray CT data with and without tension applied to specimen. Large field of view X-ray CT scans of each specimen. In-situ transilluminated white light imaging during fatigue testing.*
Data source location	*Roskilde, Denmark*
Data accessibility	*Data can be downloaded online from below links:*
	*Ex-situ X-ray CT fatigue:*http://doi.org/10.5281/zenodo.1146125
	*Applied tension (tension clamp):*http://doi.org/10.5281/zenodo.1146138
	*Transilluminated white light imaging:*http://doi.org/10.5281/zenodo.1146146

**Value of the data**

• The data shows damage initiation and progression throughout the fatigue life of a non-crimp fabric based fibre composite and establishment of 3D visualisation methods could provide additional knowledge on the damage mechanisms.
• By monitoring the damage progression in 3D in two different regions of the specimen, the data contains knowledge on how the local fibre and bundle architecture affects the damage initiation and progression, which could also be explored further e.g. by image analysis techniques.
• The ex-situ X-ray CT data can be used as a comparison base for damage progression modelling results e.g. based on the bundle structure in the large field of view scan data.
• The data where tension is applied to the specimens contains knowledge on damage features not visible in the unloaded state which is important for future studies to consider.
• Methods could be established to link the results obtained by X-ray CT and transilluminated white light imaging for increased understanding.

## 1 Data

The data published with this article includes in-situ transilluminated white light imaging (TWLI) data, ex-situ X-ray CT data, X-ray CT data with and without applied static tension during scanning for uni-directional (UD) non-crimp fabric (NCF) based fibre composites tested in tension-tension fatigue. The TWLI data shows the initiation and progression of off-axis cracks in the supporting backing fibre bundles in high resolution photographs captured during the fatigue test. For each of the 12 test specimens, a series of photos taken during the fatigue test is provided as zip files along with movies linking the images to the fatigue load cycles. The ex-situ X-ray CT data observes two regions of the same specimen in high resolution after a different number of fatigue cycles. For one region, X-ray CT is carried out after 0, 100,000, 150,000, 200,000, and 500,000 cycles, and for the second region X-ray CT is only carried out for the last three interruption points resulting in 8 data sets. The tension clamp experiments are carried out at high resolution in two regions of two different specimens tested for 100,000 and 1,000,000 cycles. X-ray CT experiments are carried out in both the loaded and unloaded state, leading to a total of 8 data sets. In addition, large field of view scans of all the 12 specimens are also provided. The X-ray CT data is provided as reconstructed data in the “.tif” format.

## 2 Experimental design, materials and methods

**Fig. 1:**
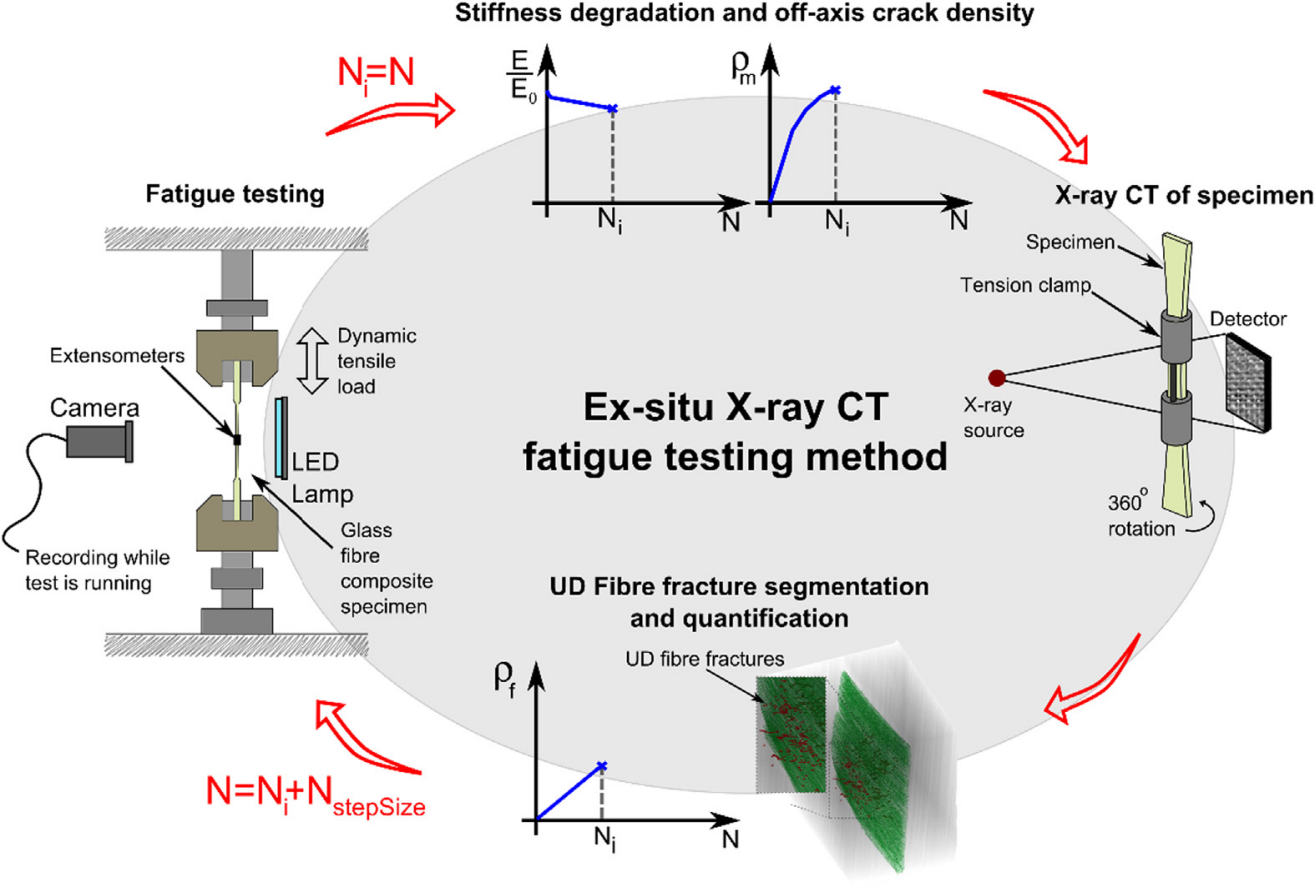
Overall idea behind ex-situ X-ray CT fatigue testing approach supplemented by TWLI and tension clamp.

The methods used to obtain the data provided with this paper are based on the idea shown in Fig. 1, which shows the general principle of the ex-situ X-ray CT fatigue testing method. To overcome some of the challenges of X-ray CT, TWLI and tension clamp experiments were also carried out as supplements. The TWLI was carried out during the fatigue test as illustrated by the camera and LED lamp in the left-hand image of Fig. 1. The tension clamp data provided with this paper is not included in the ex-situ testing loop this time, however it could be included in future work as illustrated in the left-hand image of Fig. 1 to improve the crack visibility. The below sections outline the specifics of the individual testing methods.

### 2.1 Test specimen and fatigue testing

The specimen geometry used was a modified version of the butterfly geometry optimised for testing unidirectional fibre composites [2], however with a smaller cross-section (10 × 2 mm^2^) to get higher image resolution in the X-ray CT experiments [1]. The material considered was a non-crimp glass fibre fabric based fibre composite where most of the fibres are oriented in the loading direction as unidirectional (UD) bundles. However, around 10% differently oriented (off-axis) supporting backing fibre bundles are also present to keep the UD bundles aligned during manufacturing and handling. The lay-up of the non-crimp fabric based fibre composite considered was [0/b,0/b] where ‘0’ refers to the axial fibre bundles and ‘b’ the backing fibre bundles. In the gauge section the outer backing layer was removed resulting in the layup [0/b,0], which was done to study the effect of one layer of backing in between two layers of UD fibre bundles (see also [1] for additional information).

A total of 12 tension-tension fatigue tests were carried out at strain levels ranging between 0.025% and 0.8% max strain. They were carried out at a load ratio of *R* = 0.1 and a test frequency of 5 Hz. The strain was monitored by two extensometers mounted on the sides of the specimens to not be in the way of the camera used for the TWLI. The 12 fatigue tests were all interrupted before failure (see details in Table 2 in [1]) and the one specimen used for the ex-situ X-ray CT fatigue test was interrupted for examination after 100,000, 150,000, 200,000, and 500,000 cycles.

### 2.2 Transilluminated white light imaging

Transilluminated white light imaging was carried out by lighting the specimen up from the back using an LED lamp and capturing images of the lit-up specimen using a high-resolution camera. For each specimen pen markers with 20 mm length in between were placed to be used for automatic crack counting algorithm (see [1]). Photos were captured during the fatigue test without interruptions (in-situ) and to decrease the data load photos were only captured 100 times during each cycle decade meaning that more photos are captured in the beginning of the test than after a higher number of cycles. In addition, if a sudden drop of strain of more than 1% occurred, the camera was triggered to capture additional photos. The capturing of photos was done automatically using a trigger box. The photo series captured during each fatigue test is provided as a combined.zip file and an additional file connecting the images with the cycle numbers are also provided with the data.

### 2.3 X-ray computed tomography

All X-ray CT scans were carried out using a Zeiss Xradia Versa 520 scanner with an accelerating voltage of 80 keV and a power of 7 mA. The detectors used had 2000 × 2000 pixels and binning of 2 was used combining 2 × 2 pixels resulting in ~1000 × 1000 pixels.

All the 12 test specimens were scanned over the region considered by TWLI (20 mm in length) to obtain the fibre bundle structure (large field of view). The scans were carried out with 0.4× optical magnification detector and a source to sample distance of 40 mm along with a detector-to-sample distance was 100, resulting in a pixel size of 19.5 µm in the projection images. 1601 projections were acquired during a full rotation of 360° resulting in a scan time of approximately 2 h.

**Fig. 2:**
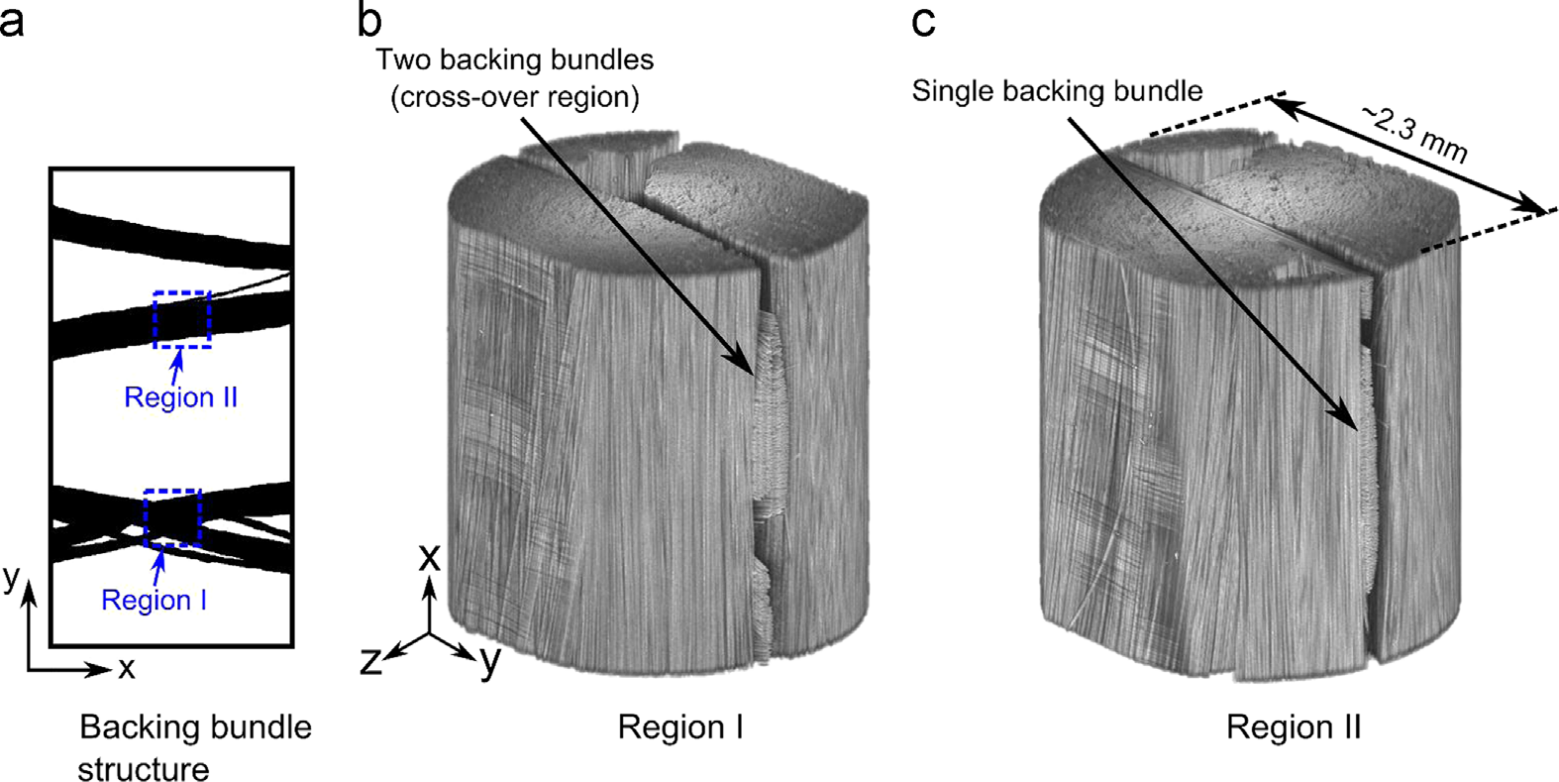
The approximate locations of the two regions considered by X-ray CT in specimen EPS06-1 are shown on top of the bundle structure in (a) and the 3D volumes obtained by X-ray CT are shown in (b) and (c) for region I and II, respectively.

**Fig. 3:**
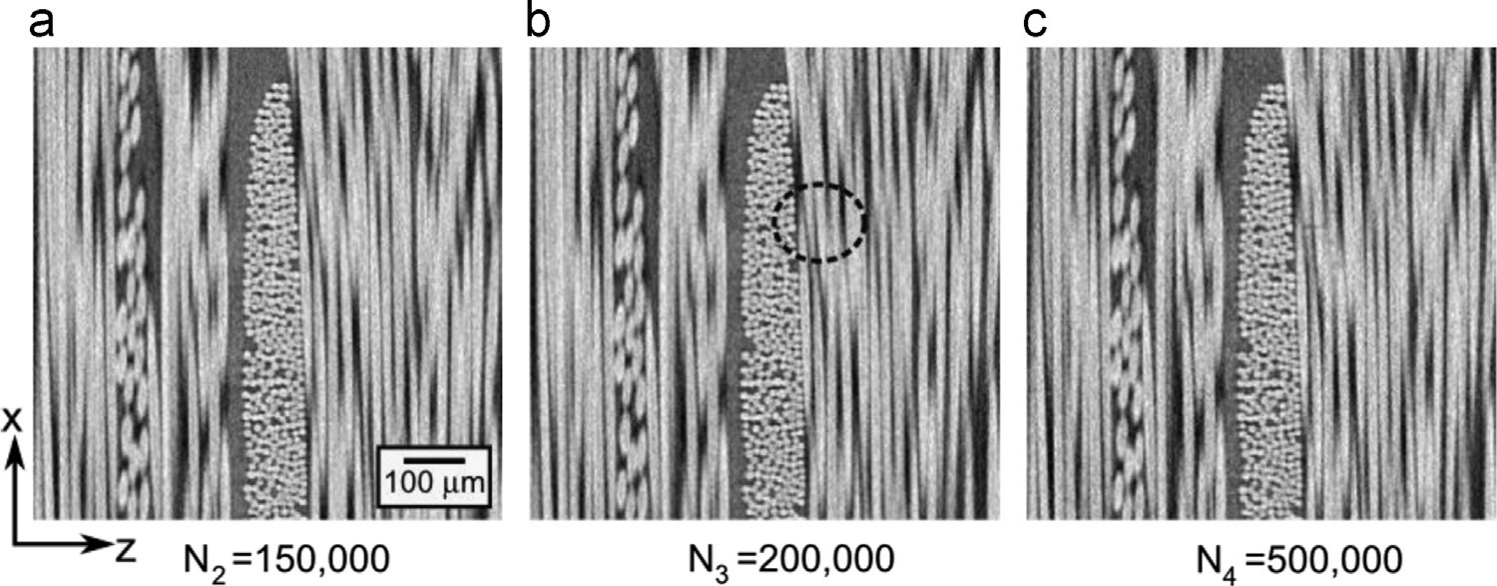
Example of 2D slice view of region II during ex-situ fatigue test.

High resolution X-ray CT with a pixel size of 2.25 µm was used for both the ex-situ study and the tension clamp experiments. For the ex-situ experiments, the source-to-sample and detector-to-sample distances were 20 mm and 40 mm respectively, and the exposure time was 2.5 s. Two regions were considered ex-situ in the specimen (EPS06-1 in Table 1) as illustrated by region I and II in Fig. 2. Region I was scanned before any testing had been done to the specimen at each of the interruption points (100,000, 150,000, 200,000, and 500,000 cycles). Region II was only scanned at the last three interruption points (150,000, 200,000, and 500,000 cycles). Fig. 3 shows an example of a 2D slice view of region II for the three interruption points. This results in 8 datasets for the ex-situ study. For clarity the data for the tension clamp have “ex-situ_” in front of the file name. The detailed scan settings are also provided as “ex-situ_info1.png” and “ex-situ_info1.png”.

Due to the presence of the tension clamp the source-to-sample and detector-to-sample distances were increased to 25 mm and 50 mm, respectively, for the tension clamp experiments and as a result the exposure time was increased to 5 s. The scans were performed with 5201 projections. Each reconstructed data set is around 2GB large. Scans were captured in two regions of two different specimens tested to 100,000 and 1,000,000 cycles respectively (region A-D in Fig. 15 in Ref. [1]). The same regions were scanned both with and without load applied. This resulted in 8 data sets for the tension clamp experiments. For clarity the data for the tension clamp have “Tensionclamp_” in front of the file name. The deleted scan settings are also provided as “Tensionclamp _info1.png” and “Tensionclamp_info1.png”. For more information on the tension clamp itself, please refer to [1].
